# Dynamic full-field optical coherence tomography: 3D live-imaging of retinal organoids

**DOI:** 10.1038/s41377-020-00375-8

**Published:** 2020-08-17

**Authors:** Jules Scholler, Kassandra Groux, Olivier Goureau, José-Alain Sahel, Mathias Fink, Sacha Reichman, Claude Boccara, Kate Grieve

**Affiliations:** 1 Institut Langevin, ESPCI Paris, PSL University, CNRS, 10 rue Vauquelin, Paris, France; 2 Institut de la Vision, Sorbonne Université, INSERM, CNRS, F-75012 Paris, France; 3 Quinze-Vingts National Eye Hospital, 28 Rue de Charenton, Paris, 75012 France; 4 Fondation Ophtalmologique Rothschild, F-75019 Paris, France; 5 Department of Ophthalmology, The University of Pittsburgh School of Medicine, Pittsburgh, PA 15213 United States

**Keywords:** Imaging and sensing, Interference microscopy, Wide-field fluorescence microscopy

## Abstract

Optical coherence tomography offers astounding opportunities to image the complex structure of living tissue but lacks functional information. We present dynamic full-field optical coherence tomography as a technique to noninvasively image living human induced pluripotent stem cell-derived retinal organoids. Coloured images with an endogenous contrast linked to organelle motility are generated, with submicrometre spatial resolution and millisecond temporal resolution, creating a way to identify specific cell types in living tissue via their function.

The comprehension of the human body and its mechanisms at the subcellular scale is still an open area of research. During the seventeenth century, the first examinations of life under the microscope were conducted directly on humans, animals and bacteria^1^. Then, at the end of the nineteenth century, cell culture studies began to replace in vivo studies, as this allows the creation of in vitro models beneficial for the comprehension of biological phenomena in different environments^2,3^. Because of the two-dimensional nature of early cell cultures, the possibilities of understanding tissues and organs as a whole were limited. Recently, three-dimensional (3D) cultures have been developed from stem cells to generate organoids that mimic a variety of tissues and serve as models of human development^4^ and disease studies^5–7^. Organoids could also serve as sources of human tissues for transplantation and as platforms for drug screening^8–10^. These self-organizing structures develop cellular compositions and architectures similar to in vivo tissues, thereby replicating biologically relevant intercellular phenomena in vitro^11,12^.

For each biological trend, optical imaging devices have been developed and optimized to image tissues, cell cultures and, recently, organoids, which are one of the most fundamental tools in biology, clinical pathology and medical diagnosis^13^. There are many challenges in imaging 3D structures: due to their relatively transparent nature, it is difficult to obtain contrast on specific structures without staining. Moreover, 3D samples require optical sectioning to discriminate the layer in focus from out-of-focus layers. In this study, we present dynamic full-field optical coherence tomography (D-FFOCT) as a technique to image retinal organoids derived from human induced pluripotent stem cells (hiPSCs)^14^, which are a major breakthrough in the study of the retina and retinal diseases^15–17^. These hiPSC-derived retinal organoids are routinely imaged with various techniques (see Supplementary Table 1). However, each of the existing methods presents major drawbacks, such as the need for fixation or mechanical sectioning, rendering the study of dynamic phenomena impossible; the need for labelling, requiring cumbersome and costly preparation; or a lack of functional contrast, indicating only cell presence and not cell health or behaviour^18–20^. Optical coherence tomography (OCT) is commonly used in biology and medicine to obtain 3D images of microstructures in tissue. OCT contrast arises from the local endogenous optical backscattering level^21^. The main drawback of traditional OCT is the trade-off between imaging depth and resolution. To increase the lateral resolution, the numerical aperture of the system must be increased. As a consequence, the depth of field decreases, and only a small layer of the sample can be imaged. Current OCT systems therefore have a lateral resolution on the order of 10 µm, which is insufficient to resolve cell structures laterally. Using an incoherent light source and a camera, time domain full-field OCT (FFOCT) is an en face variant of OCT with a higher spatial and temporal resolution than OCT in the en face plane^22^. As FFOCT acquires an entire en face plane rather than a line in the depth dimension, the numerical aperture can be arbitrarily increased without any imaging depth trade-off. Using the FFOCT experimental setup shown in Fig. 1(a) and detailed in the Methods section, a novel contrast mechanism has recently been exploited by measuring temporal fluctuations of back-scattered light in a technique called dynamic FFOCT (D-FFOCT)^23^. By revealing sub-cellular structures that have very weak back-scattering, these dynamic measurements provide contrast based on local intra-cellular motility^24,25^ with sub-micrometre resolution, and can achieve millisecond temporal resolution to study fast phenomena.

**Fig. 1:**
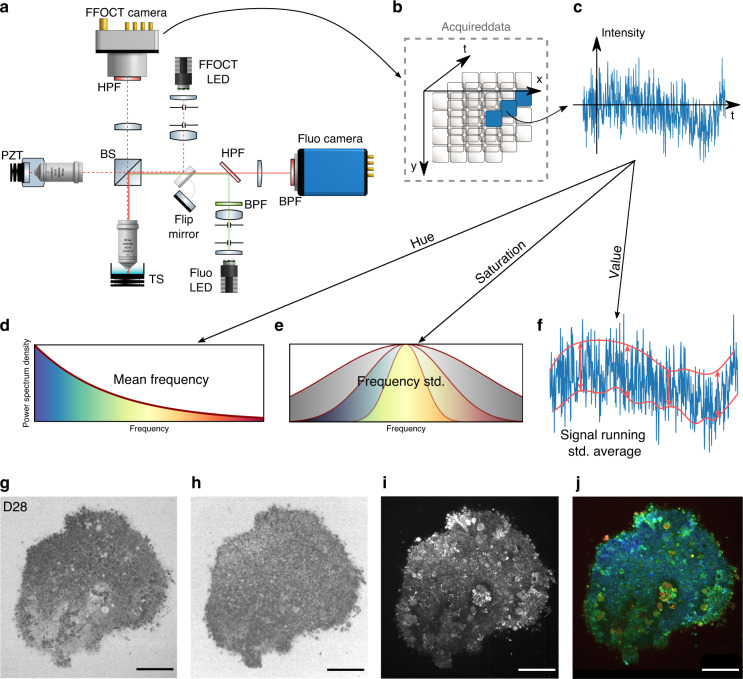
Experimental setup and post-processing schematic. **Acquisition of images a**, Full-field OCT setup combined with wide-field fluorescence microscopy (side view). PZT piezoelectric translation, TS translation stage, BPF bandpass filter, HPF high pass filter. As fluorescence is recorded in the same spectral range as that used for D-FFOCT, D-FFOCT and fluorescence images could not be recorded at the same time; for this purpose, a flip mirror was added to switch from one modality to another. **b** 3D cube of data (*x, y, t*) acquired before processing. Each time evolution of a pixel is processed independently. **c** An intensity trace is plotted for a pixel inside a living retinal organoid. **Post-processing steps d**–**f**. Dynamic images are computed in the HSV colour space. **d** Hue is computed with the mean frequency, from blue (low temporal frequencies) to red (high temporal frequencies). **e** Saturation is computed as the inverse of the frequency bandwidth; as a consequence, a signal with a broader bandwidth (e.g., white noise) appears dull, whereas a signal with narrow bandwidth appears vivid. **f** The value is computed as the running standard deviation^23^. Bottom row is a D29 retinal organoid. **g** Computation of the mean frequency (hue); **h**, frequency bandwidth (saturation); and **i**, dynamic (value) before **j**, reconstruction. Scale bar: 50 µm

In FFOCT, light coming back from the sample slice of interest interferes with light coming back from the reference mirror and is projected onto the camera (Fig. 1(a)). To compute a D-FFOCT image, a movie (typically 512 frames) of the interferogram pattern is recorded and processed (see Methods) to extract local fluctuations and render them coloured (Fig. 1(b–j)). Using the hue-saturation-value (HSV) colour space, image brightness is linked to fluctuation amplitude, whereas colour is linked to fluctuation speed, from blue (slow) to red (fast) through green (in between). By translating the sample in the axial direction to acquire a stack of planes, see Supplementary Vid. 1, a 3D volume can be reconstructed (see Methods). Alternatively, a series of dynamic images may be acquired in the same plane to follow the evolution of activity over several hours in a time-lapse fashion with a temporal resolution of up to 20 ms (see “Methods”).

A 3D reconstruction of a 28-day-old (D28) retinal organoid is depicted in Fig. 2(a), corresponding to an optic vesicle stage during retinogenesis^9,10,15,16^, along with a sub-volume in Fig. 2(b), highlighting the layered internal retinal progenitor cell organization. A cross-section is shown in Fig. 2(c), in which the elongated shape of cells is seen. A time-lapse video at 50 µm depth was acquired on the same organoid to study its temporal evolution over three hours (see Fig. 2(d) and Supplementary Video 2 for the full recording). In these acquisitions, different dynamic profiles of cells can be observed: surface cells exhibit faster dynamics than those inside the sample volume. This could be explained by the fact that at the surface of the organoid, the cells are in contact with the external environment, making them more vulnerable to change and often leading to their death. In Fig. 2(d), cells in the centre of the organoid exhibit fast and intense activity until their disappearance, possibly indicating that they are undergoing apoptosis. Evolution of cell dynamics near a clear boundary between two distinct types of cells is also visible. On one side of the boundary, cells exhibit faster and stronger dynamics, suggesting a differentiation process towards specific retinal lineages^15^, and on the other side, small rounded progenitor cells have slower activity. These two cell types are therefore distinguishable by their dynamic profile alone. Generated D-FFOCT images present a consistent colormap in which each frequency is continuously represented by the same colours; therefore, similar results are obtained for different retinal organoids at the same developmental stage. Supplementary Video 3 shows a time-lapse movie of the D28 retinal organoid shown in Fig. 2(a–c) alongside a D29 retinal organoid. The same clear boundary between distinct types of cells is present for both. By processing the data contemporaneously on the GPU using a modified version of Holovibes software^26^, an enhanced temporal resolution of 20 ms was achieved, which represents a 500-fold improvement without the need to store the raw data (up to 4 Go.s^−1^). The price paid for this improvement is the use of an alternative version of the dynamic computation, which is noise-sensitive and non-quantitative (see “Methods”). Figure 2(f–h) shows high temporal resolution (20 ms) images of a D147 retinal organoid. The typical rosette organization of retinal cells, previously described^14,15^, is visible in the centre, i.e., photoreceptors (seen from the side) in the inner part of the rosette centre with emerging outer segments (indicated by white lines on Fig. 2(h)), and other surrounding retinal cells are evident. Photoreceptor nuclei exhibit different dynamic profiles (Fig. 2(g))—either compact and uniform, inflated, or absent—which may correspond to the nuclear G0/G1, dying and M states, respectively. The gain in temporal resolution allows the study of fast biological processes such as organelles moving inside the cytoplasm (see Supplementary Video 4). A series of retinal organoids imaged by D-FFOCT at consecutive steps of development showed the gradual differentiation of retinal cell progenitors into neural cells and photoreceptors (Supplementary Fig. 2), as validated by comparison with a similar organoid series imaged with immunofluorescence on a confocal microscope^14^.

**Fig. 2:**
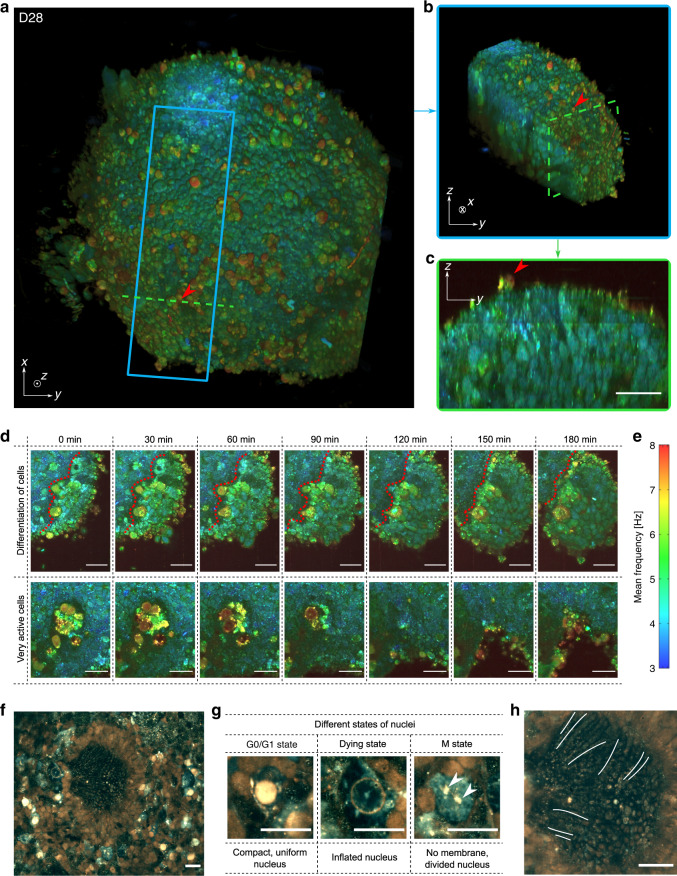
Imaging hiPSC-derived retinal organoids with D-FFOCT. **a** 3D reconstruction of the spherical D28 retinal organoid, composed of cells ~5 µm in diameter. Red arrows highlight surface cells exhibiting fast dynamics. **b** Image represents a sub-volume of **a** (blue square). **c** Image represents a cross-section in (**a**) (green dashed line) in which one can see the organization of the layers inside the retinal organoid. **d** High-magnification images of two different areas of the organoid during a 3h time-lapse acquisition: magnified images in the top row show the change in dynamic profile that could reflect a differentiation process (the boundary between the two types of cells is represented by a red dotted line); images in the bottom row show a very active zone composed of cells exhibiting fast and high dynamics, possibly undergoing apoptosis, in the centre of the organoid. **e** Colour bar of the D-FFOCT images for the 3D and time-lapse acquisitions with a consistent colormap for (**a**–**d**). High-temporal-resolution imaging performed on a D147 retinal organoid. **f** Part of the retinal organoid revealing fusiform structures corresponding to emerging photoreceptor outer segments in the centre of the rosette. **g** Magnified view of nuclei in three different states around the rosette: (i) a nucleus in a normal state with a compact, uniform shape and is very bright (i.e., exhibiting a high activity); (ii) an seemingly dying, inflated nucleus, exhibiting almost no activity; and (iii) a nucleus undergoing division with no defined nuclear membrane in the cytoplasm, and two distinct parts (white arrows) of the content of a nucleus (suggesting mitosis of the nucleus with chromosomes already divided, with the same subcellular activity level as the “normal” nucleus). **h** Magnified image of the photoreceptor outer segment-like structures imaged side-on; three of them are marked with a white line. Scale bar: 20 µm

To further validate the D-FFOCT signal origin via direct comparison between D-FFOCT and specific fluorescence labelling in the same organoids, a multimodal setup was developed that combines D-FFOCT and fluorescence channels to allow a pixel-to-pixel overlay of D-FFOCT and fluorescence images. A D29 retinal organoid was labelled with a dye targeting the nuclei of dead cells (see “Methods”). D-FFOCT images overlaid with fluorescence wide-field images are shown Fig. 3(a–d). Two fluorescent red spots are clearly visible (Fig. 3(a)) and correspond to very weak dynamic signals in the D-FFOCT image, confirming that dead cells exhibit low activity and that the contrast revealed by D-FFOCT is metabolic. These two areas are magnified in Fig. 3(c, d), and dark zones are encircled by a white dotted line. A retinal organoid was imaged with the combined D-FFOCT-fluorescence system at D126, via which a large number of differentiating photoreceptors can be detected in rosette-like configurations (Fig. 3(e, f)). A red fluorescent zone corresponding to photoreceptors is visible in Fig. 3(e), whereas in the D-FFOCT image (Fig. 3(f)), the different activity level in photoreceptors compared to surrounding cells is sufficient to provide distinction of the cell type through the dynamic signal alone, across a region that is coincident with the fluorescently labelled zone. Underlying biological processes responsible for the dynamic contrast, which has been shown to be linked to cell metabolism^23^, could include movement of organelles. Preliminary experiments suggest mitochondrial contribution, but other potential contributors, such as Golgi bodies, lysosomes, vesicles, and pigments, have not yet been discarded.

**Fig. 3:**
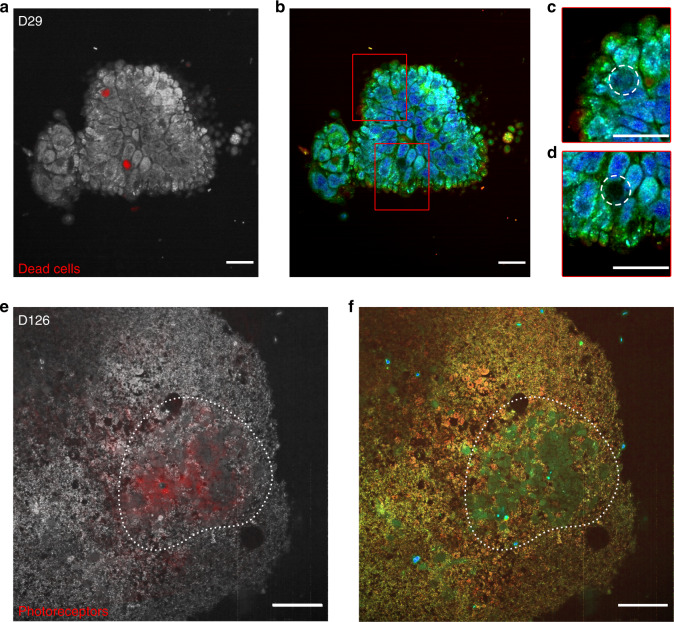
Fluorescence validation of D-FFOCT imaging. D-FFOCT images are depicted in colour (**b**–**d**, **f**), D-FFOCT images overlaid with wide-field fluorescence images are depicted in greyscale (D-FFOCT) and red (fluorescence) (**a**, **e**). **a**–**d** D29 retinal organoid labelled with dye targeting the nuclei of dead cells. **a** One can see two dead cells marked by the red spots, corresponding to the two dark zones on (**b**), the D-FFOCT image, in which there is no dynamic signal in these zones. **c**, **d** Magnified images of the two dark zones (highlighted by a white dashed line, corresponding to the two red spots of fluorescence). **e**, **f** D126 retinal organoid derived from a fluorescent cone rod homeobox (CRX) reporter iPSC line exclusively labelling photoreceptors in red (mCherry). **e** Overlaid image on which the photoreceptor fluorescence matches the blue-green cells of (**f**), the D-FFOCT image. These areas are highlighted by a white dotted line. These precursors of photoreceptors have their own particular dynamic signature, which allows them to be distinguished from the surrounding cells by D-FFOCT alone. Scale bar: (**a**–**d**) 10 µm, (**e**, **f**) 50 µm

With D-FFOCT, once installed in the culture lab under controlled environmental conditions, we anticipate being able to follow retinal organoid developmental processes that have been documented by traditional methods^14,19^, such as the quantification of cells exhibiting the same dynamic profile at multiple time points. In addition, we should also gain additional biological insights owing to the non-invasive nature of our imaging method, as it provides access to the time course of continuous processes such as progenitor cell proliferation and migration, cell type differentiation including evolution of the boundary between neural and non-neural retinal cells (Fig. 2(d)) and evolution of the organoid into layered retina. Interestingly, combining D-FFOCT and a 3D organoid-based model of retinal dystrophies due to patient-specific iPSCs offers the opportunity to understand fundamental subcellular processes leading to diseases in a way that was not previously possible.

Dynamic FFOCT imaging creates a new label-free non-invasive contrast for imaging retinal organoids. As this technique does not damage the samples, it complements and could potentially replace the imaging modalities traditionally used. D-FFOCT allows imaging of different layers at multiple depths while preserving the sample integrity, i.e., using neither exogenous labelling nor destructive methods, and is therefore suitable to follow the evolution of the same organoid at different stages of its development. The high dimensionality of the probed signals (512 interferograms per pixel) is useful for developing statistical approaches such as automated classification and clustering, and we are working towards the identification of cells via their D-FFOCT contrast alone using machine learning algorithms, with the ultimate goal of removing the need for markers entirely. The only missing part, for now, is the lack of ground truth validation data (e.g., segmented cells with labels that could be generated by fluorescence or by annotating experts), which will be a milestone in the further development of this technique.

## Methods

### Human iPSC maintenance and retinal differentiation

Two established human iPSC lines, hiPSC line-5f^17^ and fluorescent reporter AAVS1::CrxP H2BmCherry hiPSC line^27^, both derived from retinal Müller glial cells, were cultured as previously described^14^. Briefly, hiPSC lines were cultured on truncated recombinant human vitronectin-coated dishes with Essential 8TM medium (Thermo Fisher Scientific, Les Ulis, France). For retinal differentiation, adherent hiPSCs were expanded to 70–80%, and FGF-free (fibroblast growth factor) medium was added to the cultures for 2 days, followed by a neural induction period allowing retinal structures to appear. Identified retinal organoids were manually isolated and cultured as floating structures for several weeks to follow retinal cell differentiation as previously described^14,28^.

### Sample preparation for D-FFOCT imaging

Retinal organoids were placed in CO_2-_independent medium (Gibco^TM^, Thermo Fisher Scientific) in a Petri dish and kept close to 37 °C during imaging. Samples were mounted on a 3-axis translation stage under the sample arm objective and imaged directly after mounting. After imaging, each organoid was either cultured again for further D-FFOCT imaging or fixed using a solution of paraformaldehyde (PFA) for 15 min at 4 °C followed by three rinses and stored in a sucrose solution for further traditional imaging. For dead cell labelling (LIVE/DEAD Viability/Cytotoxicity Kit for Mammalian Cells, Thermo Fisher Scientific), organoids were incubated with 10 µM EthD-1 at 37 °C for 20 min before imaging and were mounted immediately after incubation and imaged within 10 min.

### Immunostaining and imaging of retinal sections

For cryosectioning, retinal organoids were fixed for 15 min in 4% PFA at 4 °C and washed in phosphate-buffered saline (PBS). Structures were incubated at 4 °C in PBS/30% sucrose (Sigma Aldrich, Saint-Quentin-Fallavier, France) solution for at least 2 h and embedded in a solution of PBS, 7.5% gelatine (Sigma Aldrich, Saint-Quentin-Fallavier, France), and 10% sucrose and frozen in isopentane at −50 °C. Ten-micrometre-thick cryosections were collected in two perpendicular planes. Sections were washed with PBS, nonspecific binding sites were blocked for 1 h at room temperature with a PBS solution containing 0.2% gelatine and 0.1% Triton X100 (blocking buffer) and then overnight at 4 °C with the primary antibodies VSX2 (goat, 1:2000, Santa Cruz Biotechnology, Clinisciences, Nanterre, France), CRX (mouse, 1:5000, Abnova, Clinisciences) and RHODOPSIN (mouse, 1:500, Merck, Guyancourt, France) diluted in blocking buffer. Slides were washed three times in PBS with 0.1% Tween and then incubated for 1 h at room temperature with appropriate secondary antibodies conjugated with either Alexa Fluor 488 or 594 (Jackson ImmunoResearch Lab., Interchim, Montluçon, France) diluted at 1:600 in blocking buffer with 4′,6-diamidino-2-phenylindole diluted at 1:1000 to counterstain nuclei. Fluorescent staining signals were captured with an Olympus FV1000 confocal microscope.

### Experimental setup

Time-domain FFOCT^22,29^ is a variant of scanning OCT^21^ in which two-dimensional en face images are captured using a CMOS camera. Three-dimensional images can be acquired and reconstructed by scanning in the depth dimension with high precision motors. This configuration, together with the use of a broad-band LED source, allows for higher axial and en face resolution than conventional OCT and can perform micrometre resolution 3D imaging noninvasively in both fresh and fixed ex vivo tissue samples. For the D-FFOCT imaging of retinal organoids, a laboratory setup was designed with a 0.5 µm lateral resolution using high-magnification water-immersion objectives (Nikon NIR APO 40x 0.8 NA), for a field of view of ~320 × 320 µm^2^. Because of the high numerical aperture, an axial resolution of 1.7 µm was also obtained by the microscope objectives in this particular configuration in which the coherence length of the source is larger than the depth of focus. The light source used for the FFOCT system was an LED with a central wavelength of 660 nm (M660L3, Thorlabs, Newton, NJ, USA). The choice of the light source for an FFOCT system is one of the most important parameters. First, it needs to be spatially incoherent to avoid cross-talk artefacts due to multiple scattering. The optical power must be high enough to image close to camera saturation to maximize the signal-to-noise ratio. The central wavelength was chosen to be as close as possible to near-infrared to maximize the penetration depth while remaining in the visible spectrum (as fluorescence measurements were done in the visible spectrum and some optical components were coated only for the visible spectrum). Finally, the LED coherence length (computed as the width at half maxima of the axial fringe pattern) was 13.28 µm in water, which allowed a robust 3D alignment between the coherence plane and the focal plane, therefore allowing a homogeneous contrast over the whole field of view. The FFOCT signal was recorded on a custom camera (Quartz 2A750, Adimec). For validation purposes, this FFOCT setup was combined with a fluorescence microscope using an LED source centred at 565 nm (M565L3, Thorlabs, Newton, NJ, USA) for excitation and filtered with a bandpass filter centred on 562 nm with a bandwidth of 40 nm (Semrock FF01-562/40-25). The emitted fluorescence was filtered with another bandpass filter centred on 624 nm (Semrock FF01-624/40-25) and then imaged using a sCMOS camera (PCO Edge 5.5). The excitation and fluorescence wavelengths were separated by a dichroic mirror at 593 nm (Semrock FF593-Di03-25).

### Data acquisition and processing

Producing each dynamic image slice required the acquisition of many (typically 512) direct images without modulating the piezo position. As opposed to static FFOCT acquisition, the measured fluctuations in D-FFOCT arise from subcellular motion. In this study, the frame rate was set to 100 Hz, which was a good trade-off between acquisition speed and signal-to-noise ratio (see the following section). For a given acquisition, we typically obtain a (1440,1440,512) tensor in which 1440 × 1440 is the number of sensor pixels and 512 is the number of recorded frames. After acquiring data, the first step is to correct for the camera frame-to-frame instability by normalizing each frame to compensate for exposure time variations. Previously, we showed colour images that were constructed by integrating signals in the Fourier domain for three frequency ranges^30^. Here, we propose a new scheme wherein colour images are computed in the HSV colour space in which, contrary to the red-green-blue (RGB) colour space, it is possible to assign a physical property to each of the three channels for quantitative visual interpretation. The idea is then to attribute a colour for each pixel depending on the characteristic time period or frequency of the dynamic signal. Each individual pixel can be thought of as a sum of subcellular random walks with a typical covariance function depending on the motion type (e.g., diffusive, hyperdiffusive). To distinguish between several dynamic profiles, we performed power spectrum analysis. Note that a correlation analysis could also be performed and gives similar results but requires more computing time. We started by computing the power spectrum density (PSD) using Welch’s method for each pixel and then used an L1 normalization on each PSD as if it were a probability distribution. Then, the hue channel, was computed as the mean frequency (which is simply the dot product between the normalized PSD and the frequency array). The values were then inverted and rescaled between 0 and 0.66 to go from blue (low frequencies) to red (high frequencies). We observed that two successive acquisitions could lead to different perceptual colour maps. We found that the lowest frequencies were slightly unstable (either due to sensor or mechanical instabilities as described in^31^). We removed this artefact by removing 3% of the lowest frequencies in the PSD. Then, the value (which corresponds to the perceived pixel intensity) was computed as the average of the temporal standard deviation with a window size of 50 samples. We saturated 0.1% of the highest value pixels to improve the contrast. For 3D stacks, the saturation threshold value was kept constant throughout the stack to obtain a consistent colour map. Finally, saturation was computed as the inverse of the normalized PSD bandwidth. As a consequence, the saturation channel carries the frequency bandwidth information. In practice, it is computed as the standard deviation of the frequencies (i.e., it corresponds to the frequency histogram width) as:

1 $$S = P.f^2 - \left( {P.f} \right)^2$$

where *P* is the normalized PSD array, *f* is the frequency array and “.” is the dot product. The saturation map is then inverted and rescaled between 0 and 0.8 to obtain a visually pleasing output. The broader the spectrum, the lower the saturation. White noise has a broader bandwidth and will therefore appear greyish instead of coloured. Finally, the dynamic image is transformed in the RGB colour space for display purposes. A (1440, 1440, 512) stack is processed in less than 10 s by the GPU (Nvidia Titan V) using MATLAB (MathWorks) and our custom software^32^, hence limiting temporal resolution by processing the data after each acquisition. To improve temporal resolution, we also used a modified version of a previous method^26^, which computes real-time dynamic images on the GPU. In this case, the algorithm used to generate dynamic images is no longer quantitative and is subject to more noise. The 3D cube of data is first Fourier transformed; the Fourier transform magnitude of each temporal signal is then integrated into three bands to reconstruct an RGB image in which the band corresponding to lower frequencies is coded in the blue channel, the band corresponding to medium frequencies is coded in the green channel and the band corresponding to high frequencies is coded in the red channel. The processing power of the GPU allowed us to process a new cube of data every 2 frames, leading to a D-FFOCT frame rate of 50 Hz in practice and a temporal resolution of 20 ms. For 3D volumes, the temporal resolution depends on the number of planes per volume. In practice, the final plane is delayed by several minutes compared with the first acquired plane. However, as the biological changes being evaluated are on time scales slower than minutes, this lag was not an issue (see Supplementary Video 2). For 3D stacks, each plane is registered during post-processing using a feature-based method (rigid registration using SIFT features and RANdom SAmple Consensus algorithms to find correct matches) to compensate for the sample lateral drift. Stacks are then interpolated in the depth dimension using bicubic interpolation to obtain a square voxel edge size of 220 nm. For 3D display purposes, a nonlocal mean filter^33^ can be applied to remove granularity.

### Choosing the frame rate for D-FFOCT

The choice of 100 Hz for the camera frame rate is the result of the optimization between the acquisition speed and the signal-to-noise ratio. For a given number of frames, the signal-to-noise ratio increases in D-FFOCT when the frame rate decreases because scattering structures have more time to move and a greater volume can be explored, therefore leading to higher phase shifts and higher intensity fluctuations on the camera. For 512 frames, the signal-to-noise ratio starts decreasing when going faster than 100 Hz, so this value was chosen to maximize both the acquisition speed and signal-to-noise ratio.

### Correcting for sample drift

Due to thermal effects, we observed a slow mechanical drift (on the order of 5 µm.h^−1^), which could prevent us from measuring dynamic images in the same plane over several hours (axial resolution is 1.7 µm). To compensate for this drift, we developed a correlation-based method called the plane locking procedure^25^. This procedure was utilized when the cross-correlation between the current image and the target image was below a threshold (typically between 0.2 and 0.4). In this case, FFOCT images were acquired over an axial extent of 10 µm, with 0.5 µm steps using the sample translation stage and were then cross-correlated with the target image. The sample was then axially translated to the position corresponding to the maximum of the cross-correlation, which was typically between 0.5 and 0.8. After each plane correction procedure, a new FFOCT image was chosen as the target for the next correction to account for evolution in the sample position.

### Combining FFOCT and fluorescence microscope channels

The FFOCT and fluorescence cameras do not share the same sensor size and resolution, so to construct overlays, FFOCT images were registered onto fluorescence images using a projective transformation. The projective transformation was calibrated using a deformation target (R1DS1N, Thorlabs, Newton, NJ, USA) before experiments. The final overlays were constructed in RGB colour space, the value channel corresponding to dynamic amplitude was inserted into every channel (R, G, and B), and a fluorescence image was added only to the R channel. In this way, the dynamic image appeared in greyscale with the fluorescence superimposed in red.

## Supplementary information

Supplementary information and files

Supplementary Video 1

Supplementary Video 2

Supplementary Video 3

Supplementary Video 4

Supplementary Video 5

Supplementary Video 6

## Data Availability

The study data are available from the corresponding author upon request.

## Code availability

The control and acquisition software is available at https://github.com/JulesScholler/FFOCT.
