# Supplementary material for: Dynamic full-field optical coherence tomography: 3D live-imaging of retinal organoids
Source: Light Sci Appl. 2020 Aug 17;9:140. doi: 10.1038/s41377-020-00375-8 (PMC7429964; doi:10.1038/s41377-020-00375-8)
Supplement: Supplementary file 1 — Supplementary information and files [file 41377_2020_375_MOESM1_ESM.docx]

**Integrated supplementary information for**

# Dynamic full-field optical coherence tomography: 3D

# live-imaging of retinal organoids

Jules Scholler^1^*^,^*^†^, Kassandra Groux^1^*^,^*^†^, Olivier Goureau^2^, José-Alain Sahel^2^*^,^*^3^*^,^*^4^*^,^*^5^, Mathias Fink^1^,

Sacha Reichman^2^, Claude Boccara^1^ and Kate Grieve^2^*^,^*^3^*^,^*^∗^

^1^Institut Langevin, ESPCI Paris, PSL University, CNRS, 10 rue Vauquelin, Paris, France

^2^Institut de la Vision, Sorbonne Université, INSERM, CNRS, F-75012, Paris, France

^3^Quinze-Vingts National Eye Hospital, 28 Rue de Charenton, Paris, 75012, France

^4^Fondation Ophtalmologique Rothschild, F-75019 Paris, France

^5^Department of Ophthalmology, The University of Pittsburgh School of Medicine, Pittsburgh, PA 15213, United States

^†^These authors contributed equally to this work

^∗^kategrieve@gmail.com


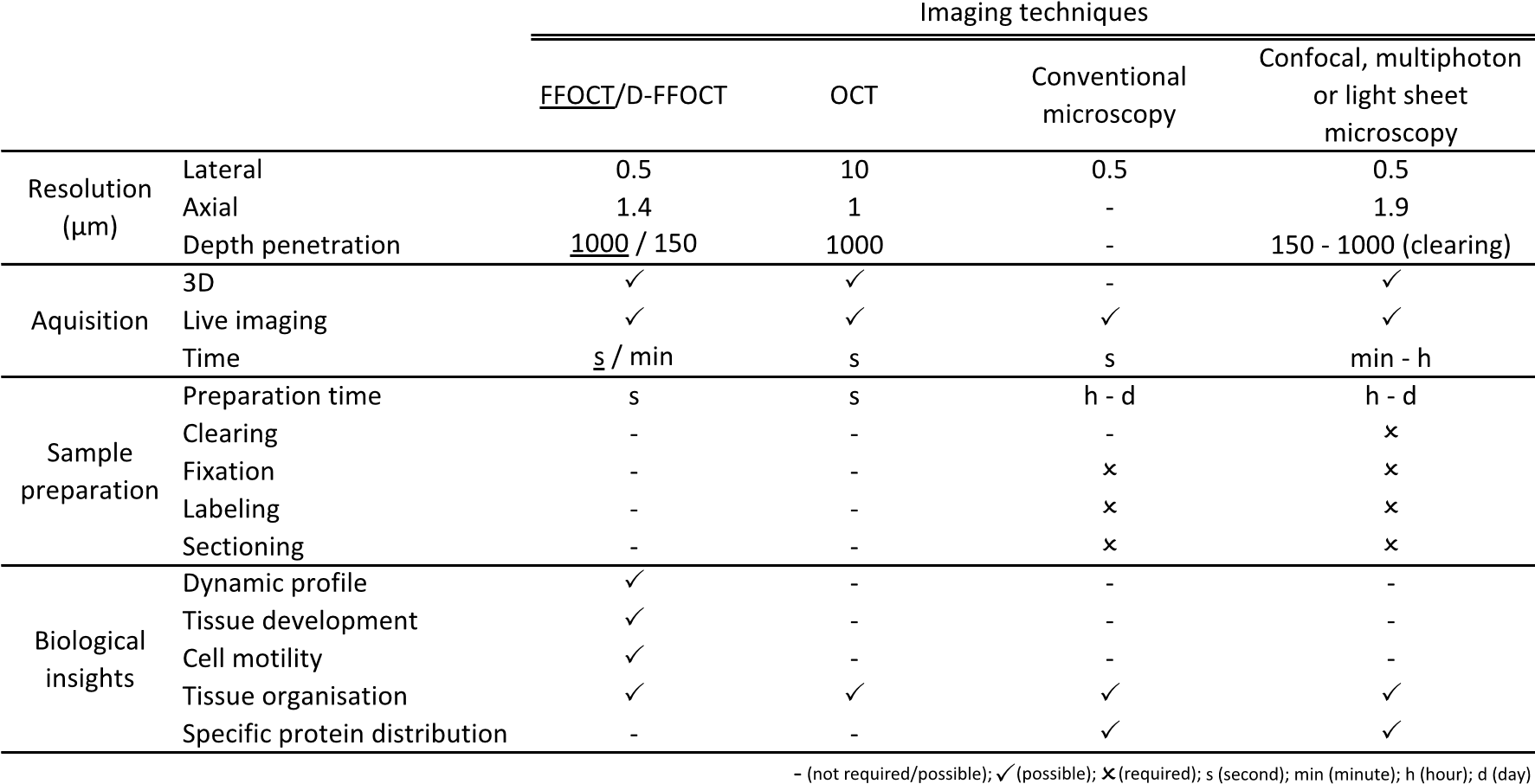


Table 1: Comparison of the different techniques that can be used to image hiPSC-derived retinal organoids. Lateral resolution computed for the same microscope objective (Nikon NIR APO 40x 0.8 NA) is diffraction-limited for all methods aside from OCT, wherein the need for a large depth of field limits the lateral resolution to 10 *µm*. Axial resolution in (D-)FFOCT is determined by the depth of field of the objective rather than the coherence length in the current configuration as high NA objectives are used (i.e., as the depth of field is smaller than the coherence length, the axial resolution is limited by the numerical aperture). Confocal, multiphoton, and light sheet immunohistochemistry methods achieve the same axial resolution at shallow depths as (D-)FFOCT but usually require invasive tissue clearing procedures to maintain the resolution at greater depths. The lower penetration depth of D-FFOCT than that of FFOCT comes from the low reflectivity of the moving organelles [31] that produce the dynamic signals. No method can currently penetrate the full organoid depth with submicrometre resolution without sectioning. However, imaging of the entire organoid is not always necessary because with an imaging depth of 150 *µm*, part of the organoid that is representative of the entire structure can be successfully imaged even in the largest organoids, thanks to their spherical organization. Should 3D imaging of entire organoids prove worthwhile, aberration correction methods could be utilized with FFOCT to reach greater penetration depths, combined with organoid rotation and additional postprocessing. Immunohistochemistry methods rely either on staining, which adds specific colour contrast and in which the sample is mechanically sliced to provide sectioning (immunohistology), or with targeted dyes, wherein various techniques (confocal illumination/detection, light-sheet illumination, etc.) provide optical sectioning. Relying on exogenous contrasts for imaging at specific time points greatly limits the structures or events that can be seen in samples and ultimately leads to the destruction of the sample, as these techniques all require sectioning, clearing or fixation steps that preclude live imaging and, therefore, monitoring of the development of the same sample over a long period of time. D-FFOCT is not only a unique technique that combines high spatial and temporal resolution but also a non-destructive approach that provides the possibility of following the development of the same organoids over time throughout development. This process could be automated by integrating the D-FFOCT setup directly inside a cell culture incubator under a *CO*_2_ atmosphere. D-FFOCT can therefore offer new biological insights such as cellular motility, tissue development and organization and dynamic profile evolution and comparison.


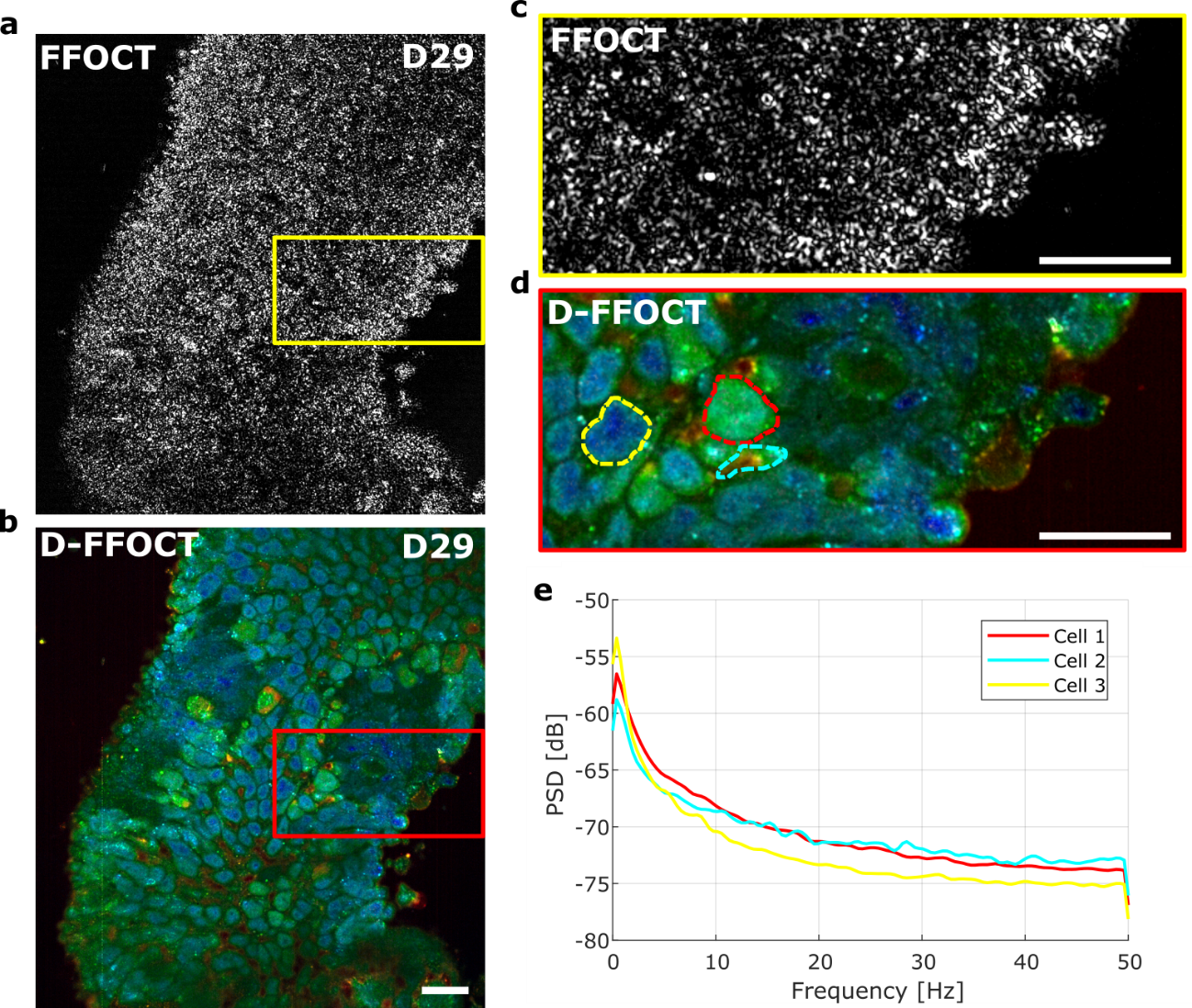


Figure 1: Comparison between FFOCT and D-FFOCT images on a D29 organoid. (a) FFOCT image of a D29 retinal organoid with (b) the corresponding D-FFOCT image. The FFOCT image was acquired using a 2-phase bucket scheme using a piezoelectric actuator to move the reference mirror [25] and extract the interferometric signal. 256 FFOCT amplitude images (corresponding to a total of 512 acquired images with 256 images for each phase) were averaged to obtain the final output. The D-FFOCT image was acquired with 512 direct images (without using piezoelectric modulation) and exhibits much higher contrast on cells. (c) Magnification of the FFOCT image (a) with the corresponding magnification of the D-FFOCT image (d). The mean power spectrum density was computed using Welch's method on the 3 cells drawn with the dashed line in (d) on graph (e). Scale-bar: 20 *µm*.


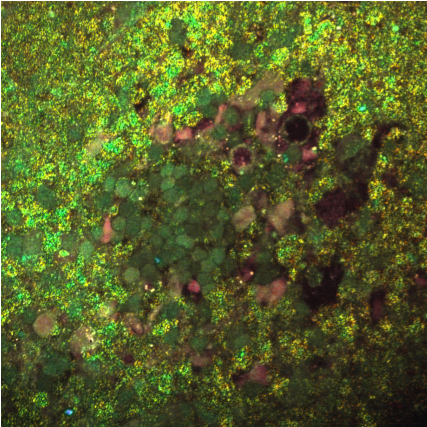

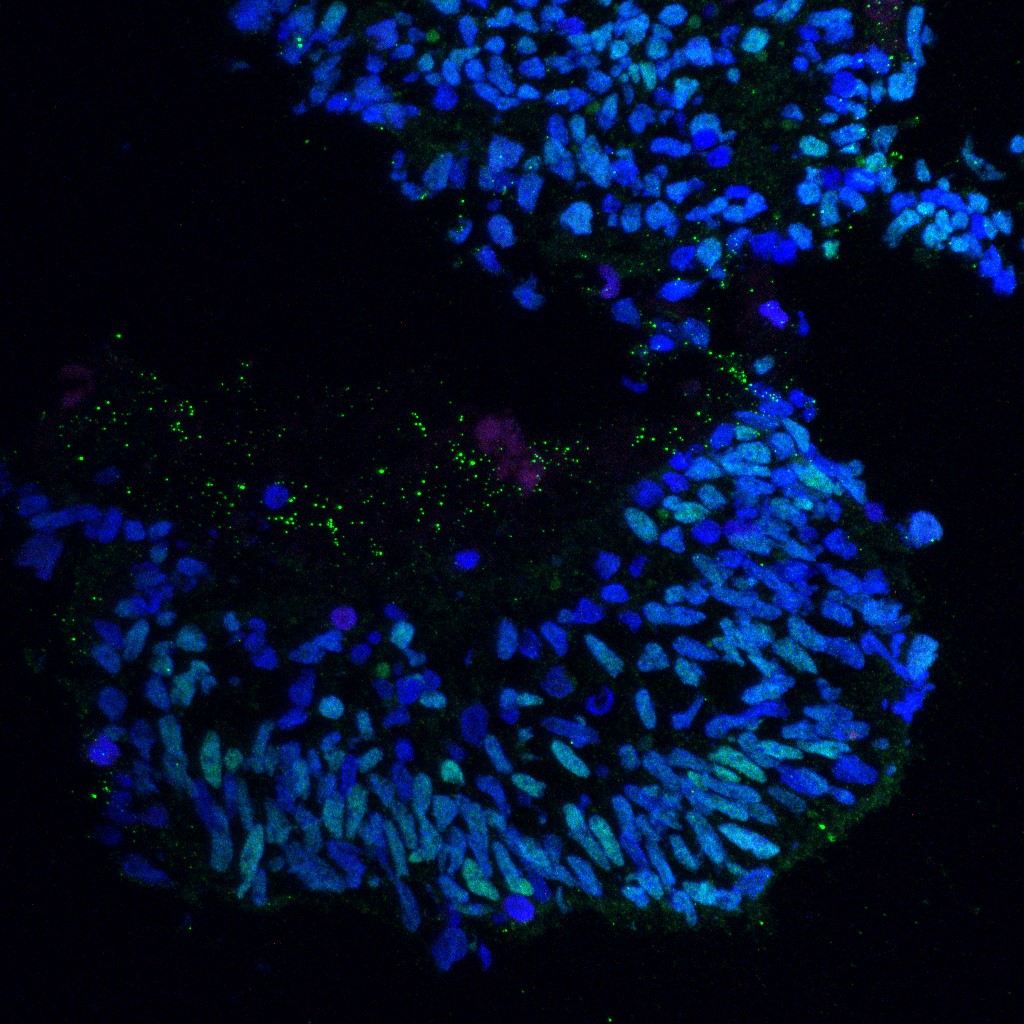

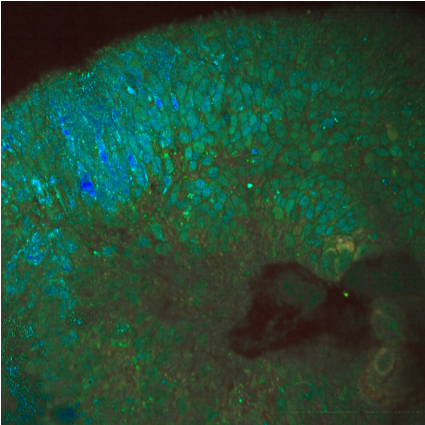

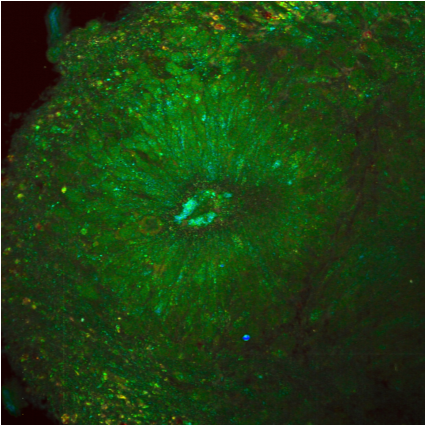


**D35**

**D51**

**D177**


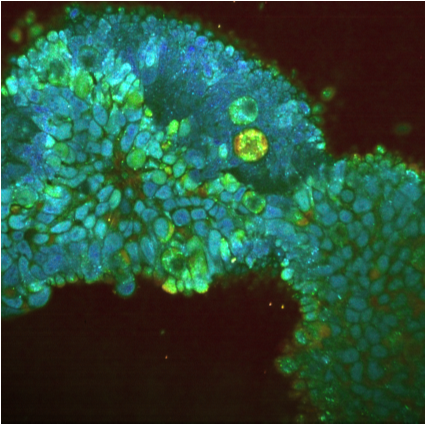

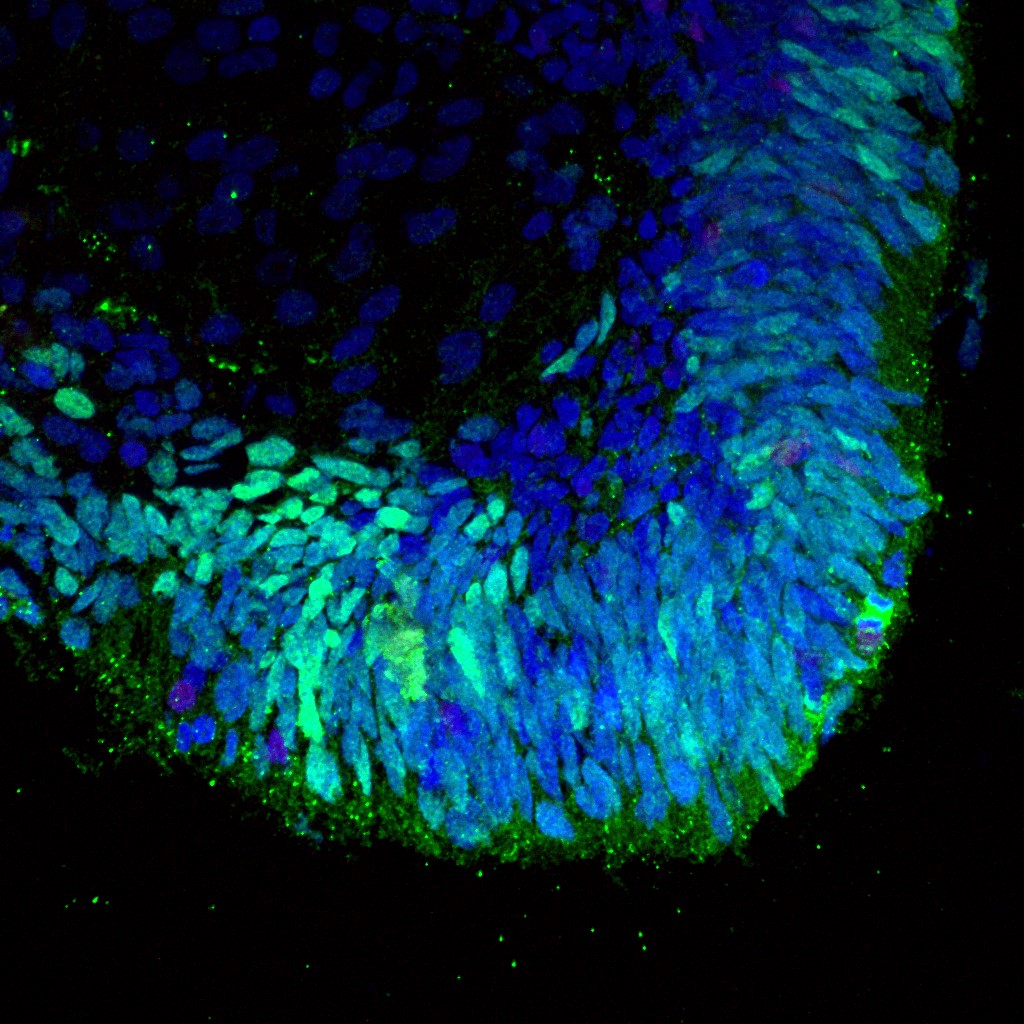

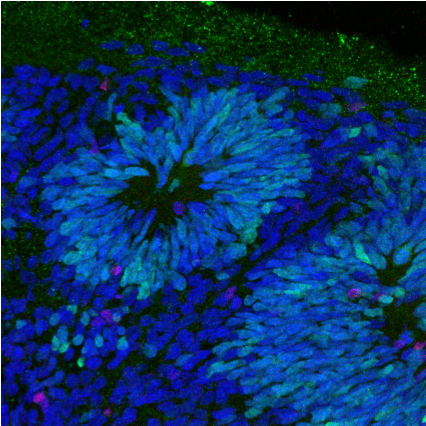


**D28**

**D35**

**D50**

**VSX2**

**VSX2**

**VSX2**


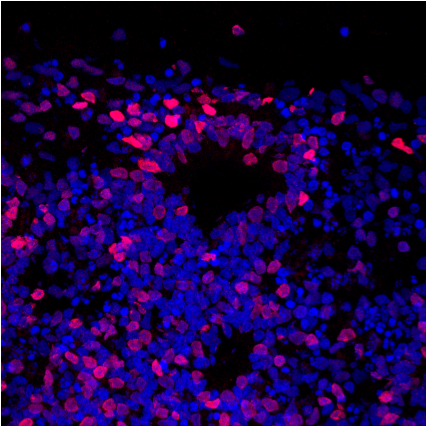


**D177**

**CRX**

**D29**

**mCherry**

**mCherry**

**mCherry**

Figure 2: D-FFOCT imaging (top) and immunohistochemistry images acquired with confocal microscopy (bottom) of distinct retinal organoids at four equivalent stages of development. On D29 and D35, organoids are mainly composed of multipotent retinal progenitor cells expressing the Visual System Homeobox 2 (VSX2) transcription factor (green). At D51, rosette formation observed by D-FFOCT was confirmed by immunohistochemistry with differentiation of progenitor cells (red dotted lines). Photoreceptors can be identified in immunohistochemistry by CRX expression in rosette structures at D177 and are seen in DFFOCT as blue-green cells in the rosette centre (white arrows). In addition, the D-FFOCT image shows putative inner retinal neurons (identified due to their position around the edge of the rosette [15]) in red (yellow arrows). Nuclei are counterstained with DAPI (blue). The field of view of D-FFOCT images has been reduced to correspond to the field of view of the confocal images. Scale bar: 50 *µm*.


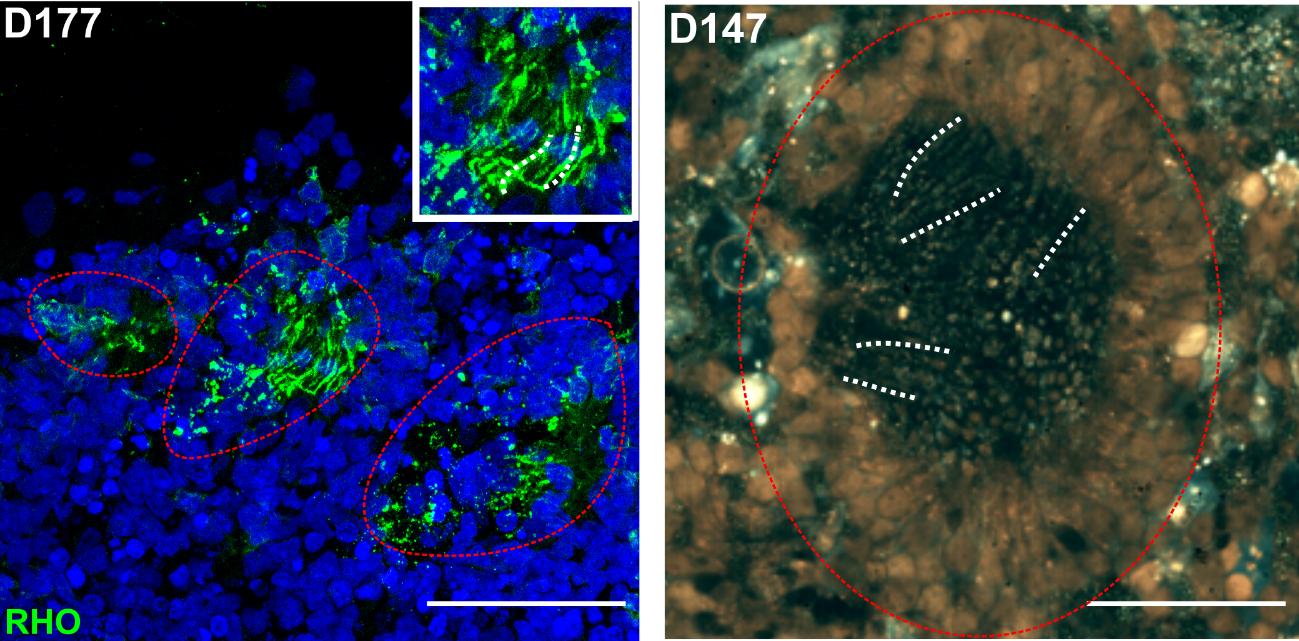


Figure 3: Immunohistochemistry image (left) acquired with a confocal microscope of a D177 retinal organoid wherein photoreceptor outer segments containing rhodopsin (green fluorescent label) are visible inside rosettes (red dotted lines). The magnified image in the top right corner highlights photoreceptor outer segments (green filaments, annotated with white dotted lines). Nuclei are counterstained with DAPI (blue). D-FFOCT image (right) of a similar retinal organoid at D147 showing a rosette (red dotted line) containing photoreceptor outer segments (white dotted lines), similar to those in the immunohistochemistry image. Rosette sizes can vary greatly and happen to be smaller in the organoid on the left than in the organoid on the right. The D-FFOCT field of view has been cropped to match the immunohistochemistry image; see Supplementary Vid. 4 for the whole field of view. Scale bar: 50 *µm*.

**Supplementary files information**

**Video 1:**

Depth stack of the D28 hiPSC-derived retinal organoid shown in Fig. 2(a-c). Planes from top 0 *µm* to approximate center of the organoid 101 *µm*, in 1 *µm* steps are shown revealing the internal structure of the organoid.

**Video 2:**

Time-lapse video with 1 min temporal resolution of the evolution of the D28 hiPSC-derived retinal organoid shown in Fig. 2(d). Video shows the evolution of the whole organoid over three hours of imaging, with three magnifications corresponding to the zones depicted in Fig. 2(d).

**Video 3:**

Time-lapse video with 1 min temporal resolution of the evolution of the D29 hiPSC-derived retinal organoid shown in Supplementary Fig. 2. Video shows the evolution of the whole organoid over three hours of imaging.

**Video 4:**

Time-lapse of the D29 hiPSC-derived retinal organoid and of the D28 hiPSC-derived retinal organoid. Both organoids exhibit the same consistent colormap and structures demonstrating that D-FFOCT can reliably images different samples at different times consistently.

**Video 5:**

Time-lapse video with 1 min temporal resolution of the evolution of a D42 hiPSC-derived retinal organoid. Video shows the evolution of the whole organoid over three hours of imaging, especially the behaviour in a rosette.

**Video 6:**

Time-lapse video with 5 seconds temporal resolution of the evolution of a D147 hiPSC-derived retinal organoid with zoomed areas. Zoomed areas have a temporal resolution of 1 second and highlight the sensitivity of D-FFOCT to dynamic phenomena. The colors are computed like the images of Fig. 2(f-h).
